# Abdominal Aortic Occlusion and the Inflammatory Effects in Heart and Brain

**DOI:** 10.1155/2023/2730841

**Published:** 2023-11-23

**Authors:** Jun Xu, Sijie Li, Alexandra Wehbe, Xunming Ji, Yong Yang, Yu Yang, Linhui Qin, Feng-Yong Liu, Yuchuan Ding, Changhong Ren

**Affiliations:** ^1^Beijing Key Laboratory of Hypoxia Translational Medicine, Xuanwu Hospital, Center of Stroke, Beijing Institute of Brain Disorder, Capital Medical University, Beijing 100053, China; ^2^Department of Neurosurgery, Wayne State University School of Medicine, Detroit, MI 48201, USA; ^3^Harvard T. H. Chan School of Public Health, Boston, MA 02115, USA; ^4^School of Chinese Medicine, Beijing University of Chines Medicine, Beijing 100029, China; ^5^Department of Interventional Radiology, Senior Department of Oncology, Fifth Medical Center of PLA General Hospital, Beijing, China

## Abstract

**Background:**

Abdominal aortic occlusion (AAO) occurs frequently and causes ischemia/reperfusion (I/R) injury to distant organs. In this study, we aimed to investigate whether AAO induced I/R injury and subsequent damage in cardiac and neurologic tissue. We also aimed to investigate the how length of ischemic time in AAO influences reactive oxygen species (ROS) production and inflammatory marker levels in the heart, brain, and serum.

**Methods:**

Sixty male C57BL/6 mice were used in this study. The mice were randomly divided into either sham group or AAO group. The AAO group was further subdivided into 1–4 hr groups of aortic occlusion times. The infrarenal abdominal aorta was clamped for 1–4 hr depending on the AAO group and was then reperfused for 24 hr after clamp removal. Serum, hippocampus, and left ventricle tissue samples were then subjected to biochemical and histopathological analyses.

**Results:**

AAO-induced I/R injury had no effect on cell necrosis, cell apoptosis, or ROS production. However, serum and hippocampus levels of malondialdehyde (MDA) and lactate dehydrogenase (LDH) increased in AAO groups when compared to sham group. Superoxide dismutase and total antioxidant capacity decreased in the serum, hippocampus, and left ventricle. In the serum, AAO increased the level of inducible nitric oxide synthase (iNOS) and decreased the levels of anti-inflammatory factors (such as arginase-1), transforming growth factor- *β*1 (TGF-*β*1), interleukin 4 (IL-4), and interleukin 10 (IL-10). In the hippocampus, AAO increased the levels of tumor necrosis factor (TNF-*α*), interleukin 1*β* (IL-1*β*), interleukin 6 (IL-6), IL-4, and IL-6, and decreased the level of TGF-*β*1. In the left ventricle, AAO increased the level of iNOS and decreased the levels of TGF-*β*1, IL-4, and IL-10.

**Conclusions:**

AAO did not induce cell necrosis or apoptosis in cardiac or neurologic tissue, but it can cause inflammation in the serum, brain, and heart.

## 1. Introduction

There are several clinical circumstances that can lead to abdominal aortic occlusion (AAO)-induced ischemia/reperfusion (I/R) injury, such as perivascular surgery, repair of abdominal aortic aneurysms or dissections, acute abdominal aortic thromboembolism, emergency trauma surgery, or balloon occlusion therapy after abdominal blood loss [1, 2]. Even though such clinical phenomena are known to cause ischemic and ischemia/reperfusion injuries in organs directly affected by reduced blood supply from the aorta (kidneys, gastrointestinal tract, lower limb muscles), other remote organs may also be affected, such as the heart and lung [3, 4]. Due to systemic inflammation and multiorgan dysfunction that occurs during the reperfusion phase, AAO-induced I/R injury carries high rates of morbidity and mortality [5].

Many factors influence the severity of distant organ injury caused by I/R injury after AAO, such as severity of hemodynamic injury, tissue susceptibility to ischemia, and duration of tissue ischemia [6]. A previous study using a rat AAO model (ischemia for 60 min followed by 2 hr of reperfusion) found alveolar congestion and the presence of neutrophils and leukocytes throughout the lung and tubular damage, the loss of brush border, and interstitial edema in renal tissue on histopathological examination [7]. However, in this same study, no pathological changes were found within cardiac tissue [7]. It has been shown that suprarenal AAO for 30 min results in cellular vacuolization, focal interstitial edema, and hemorrhagic infiltration in cardiac tissue [8]. Furthermore, prior studies have shown that neurologic tissue is intolerant to ischemia, and the hippocampus in particular is especially sensitive to ischemic conditions [9]. Renal artery occlusion for 45 min has been reported to induce distal neurologic tissue necrosis and glial cell proliferation [10]. The brain has the highest oxygen demand among all organs in the body, thus making it the most sensitive to hypoxia/ischemia [10]. However, the effects of infrarenal AAO on the heart and brain are not well understood.

The primary aim of this study was to investigate the effects of infrarenal AAO and reperfusion on the cardiac and neurologic tissue in mice.

There are numerous factors responsible for systemic complications from AAO, including oxidative stress, neutrophil infiltration, various proinflammatory cytokines, endothelial injury, and disruption of the ion transport system in the cell membrane [7, 11]. One crucial underlying mechanism of injury is the overproduction of reactive oxygen species (ROS) and proinflammatory molecules, amplifying the body's inflammatory response [12]. ROS promotes tissue injury through endothelial activation, cytokine release, and leukocyte activation [13]. An oxidant-mediated injury can be demonstrated by measuring tissue malondialdehyde (MDA) and lactate dehydrogenase (LDH) levels that occur in tissues [14]. Cells are protected from ROS by endogen antioxidant enzymes, such as superoxide dismutase (SOD), glutathione reductase, catalase, and glutathione peroxidase. Therefore, levels of SOD and total antioxidant capacity (TAC) can be measured to evaluate antioxidant capacity [15]. During I/R, numerous proinflammatory factors, such as inducible nitric oxide synthase (iNOS), tumor necrosis factor (TNF-*α*), interleukin-1*β* (IL-1*β*), and interleukin-6 (IL-6), were rapidly released [12]. Upregulation of proinflammatory factors can further aggravate tissue damage [8]. However, anti-inflammatory factors such as arginase-1 (Arg-1), transforming growth factor-*β*1 (TGF-*β*1), interleukin 4 (IL-4), and interleukin 10 (IL-10) also reflect the extent of tissue damage [16]. These parameters were used to quantify the effects of varying lengths of ischemia from AAO on ROS and inflammation levels in the heart, brain, and serum in mice.

## 2. Materials and Methods

### 2.1. Animals

Eight-week-old male C57BL/6 mice weighing 20 g were purchased from Vital River Laboratory Animal Technology Co. Ltd. They were housed in a temperature-controlled room (23 ± 1°C) and a 12 hr light–dark cycle-controlled with free access to food and water [12]. All animal experiments in this study were approved and reviewed by the Institutional Animal Care and Use Committee of Capital Medical University on October 14, 2021 (date and number: 2021/255) and complied with the Guide for the Care and Use of Laboratory Animals.

### 2.2. Abdominal Aortic Ischemia/Reperfusion Injury Model

**Figure 1:**
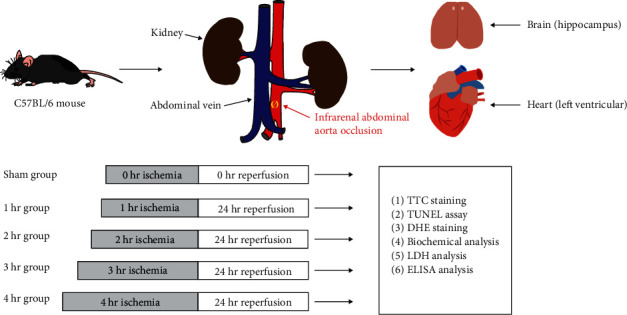
Schematic presentation of the experimental design. In this study, male C57BL/6 mice were used in this study. The mice were randomly divided into either sham group or AAO group. In the sham group, only a laparotomy was performed. In the AAO groups, the abdominal aorta under left renal 0.5 cm was occluded with sutures for 1–4 hr, followed by 24 hr of reperfusion. AAO, abdominal aorta occlusion; I/R, ischemia/reperfusion.

In this study, the AAO model was used to investigate I/R injury in the brain and heart [12]. All mice were randomly divided into five groups: the sham group and I/R groups with 1–4 hr of AAO (*n* = 7 per group). Animals were anesthetized with 1.5% enflurane, 30% O_2_, and 68.5% N_2_O. The abdomen was then explored through a midline incision after shaving and disinfection. In the sham group, only a laparotomy was performed. In the I/R group, the abdominal aortic under left renal 0.5 cm was occluded with sutures for 1–4 hr, followed by 24 hr of reperfusion [17]. The middle cerebral artery occlusion (MCAO) model was performed as described in a previous study [18]. A laser speckle contrast imager (PSI system, Perimed Inc.) was used to evaluate whether the nylon filament silicon tip was inserted into the right MCA. During the operation, mice body temperature was maintained by a temperature-controlling pad (Harvard Apparatus, MA, USA). At the end of the procedures, the animals were sacrificed after blood sampling, and then neurologic and cardiac tissue samples were obtained from all mice. Experimental design was shown in Figure 1.

### 2.3. Triphenyl Tetrazolium Chloride (TTC) Staining

The infarct volume of brain and heart tissue induced by I/R was measured by TTC staining at 24 hr after AAO. The tissue was sectionally cut into 2 mm-thick coronal slices and then TTC staining was performed according to the manufacturer's introductions (Sigma, Cat. no.: 362883) [18]. In brief, the slices were incubated in phosphate buffer containing 1% sodium chloride triphenyl tetrazolium (TTC in 0.2 M Tris buffer, pH 7.4) (Sigma-Aldrich, St. Louis, MO, USA) for 20 min at 37°C and soaked in 4% paraformaldehyde (Sigma-Aldrich, St. Louis, USA) for 24 hr. Formazan, a red-colored compound, was produced by the reaction of TTC with dehydrogenase in normal tissues. Thus, viable cells were stained red and necrotic cells remained white [19]. All experimental results were photographed and the infarct area was analyzed with ImageJ software (National Institutes of Health, Bethesda, MD, USA).

### 2.4. TUNEL Assay

Cell apoptosis within neurologic and cardiac tissue was detected by the terminal transferase-mediated dUTP nick-end labeling (TUNEL) assay kit (Roche, Mannheim, Germany, Cat. no.: 11684795901) [20]. Neurologic and cardiac tissue samples were soaked in Tissue-Tek® O.C.T. compound (SAKURA, Japan, Cat. no.: 4583) and frozen immediately in liquid nitrogen. The tissue was cut into 10-*μ*m frozen sections for TUNEL staining according to the supplier's instructions to detect the DNA fragments produced during apoptosis. The samples were treated with encapsulating solution containing 4′,6-diamidino-2′-phenylindole (DAPI) and further observed under a confocal microscope. The positive cells are labeled green and the nuclei blue. Five sections were randomly selected (×400) of each piece for photographing and analyzed with ImageJ software. The apoptosis-positive cell was calculated as the number of TUNEL-positive cells divided by the total number of cells [21].

### 2.5. Dihydroethidium (DHE) Staining

To evaluate the production of ROS in neurologic and cardiac tissue, the invitrogen dihydroethidium (DHE) kit (Thermo Fisher Scientific, Waltham, MA, USA, Cat. no.: D23107) was used for detection [22]. DHE staining was performed according to the manufacturer's protocols. In brief, 10-*μ*m-thick frozen tissue sections were incubated with DHE solution for 30 min at 37°C, and then treated with encapsulating solution containing DAPI. The positive cells were labeled red and the nuclei labeled blue. Five sections were randomly selected (×400) of each piece for photographing and analyzed with ImageJ software. The ROS rate (%) was calculated as the number of DHE-positive cells divided by the total number of cells.

### 2.6. Biochemical Analysis

#### 2.6.1. Tissue Homogenization

Hippocampus and left ventricular tissue samples were immediately frozen in liquid nitrogen and stored at −80°C. When the measurement was started, all tissue samples were weighed and washed in ice-cold phosphate-buffered saline at 1 : 9 ratio, then homogenized with an electric homogenizer for 10 min, and centrifuged in 4°C at 12,000 *g* for 5 min. The tissue supernatant was taken for MDA, SOD, and TAC assays and stored at −80°C.

#### 2.6.2. Determination of Protein Concentration

The total protein concentration of serum and tissue samples were quantified by bicinchoninic acid assay (BCA) which was performed using APPLYGEN BCA protein assay kit (Beijing, China, Cat. no.: P1511-1). Bovine serum albumin was used as a protein standard. The absorbance at 562 nm (A562) of all samples was measured with InnoScan 300 Microarray Scanner (Innopsys, France), and concentration was calculated using a standard curve. All samples were stored at −80°C for later use.

#### 2.6.3. Determination of MDA, SOD, and TAC

In tissue and serum samples, MDA (Lipid Peroxidation MDA assay kit, Cat. no.: S0131), SOD (SOD assay kit with WST-8, Cat. no.: S0101), and TAC levels (T-AOC assay kit, Cat. no.: S0119) were determined in accordance with the Beyotime Biotechnology instruction.

### 2.7. Lactate Dehydrogenase (LDH) Activity Assessment

The LDH activity of tissue and serum samples was determined by Nanjing Jiancheng LDH kit (Nanjing, China, Cat. no.: A020) for evaluating I/R injury [23]. According to the instruction provided by the manufacturer, we measured the optical density at 450 nm (A450) to confirm the activity of LDH.

### 2.8. ELISA Analysis

The levels of TNF-*α*, IL-1*β*, IL-6, TGF-*β*1, IL-4, and IL-10 were determined by ELISA kits from NeoBioscience Technology (Beijing, China). The levels of iNOS and Arg-1 were determined by ELISA kits from Enzyme-linked Biotechnology (Shanghai, China). According to the instruction provided by the manufacturer, measure the absorbance at 450 nm (A450) of all samples and bring it into a standard curve for calculation. All samples were measured in duplicate.

### 2.9. Statistical Analysis

All data are expressed as mean ± standard error of the mean (SEM) from each independent experiments. Statistical analyses were performed with GraphPad Prism version 6.0 (GraphPad Software, San Diego, CA, USA). Greater or equal to three groups were analyzed using one-way ANOVA analysis followed by Tukey's honestly significant difference test; the comparison between two groups was analyzed by Student's *t*-test. For all analyses, *p* value of <0.05 was considered significant.

## 3. Results

### 3.1. The Effect of AAO on Ischemia Injure of Brain and Heart

**Figure 2:**
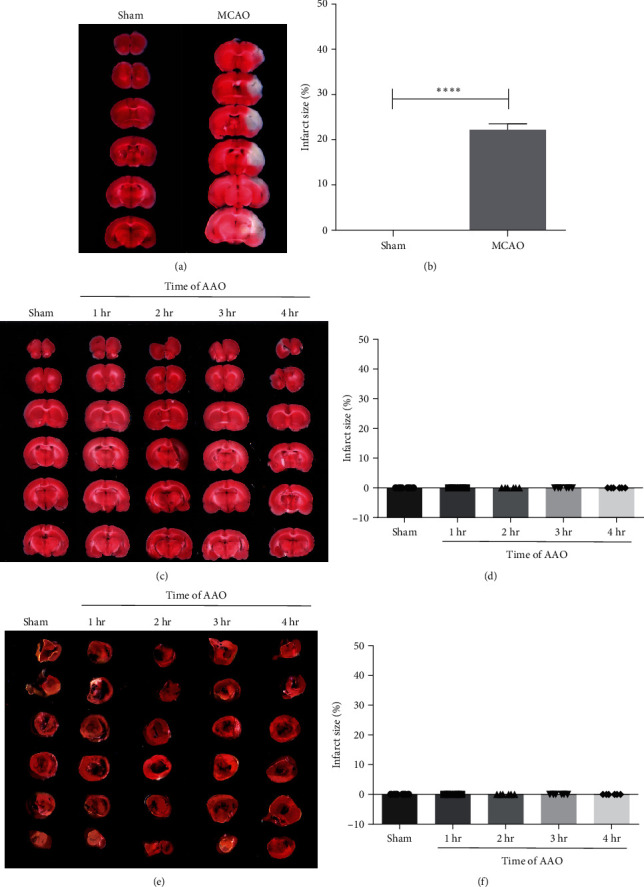
The effect of AAO on infarct size of brain and heart in mice. The I/R induced infarct volume in different groups was determined by TTC staining. (a) Representative TTC-stained images of brain slices in MCAO model. (b) Quantification of cerebral infarct size at 24 hr after MCAO. (c) Representative TTC-stained images of brain slices in AAO model. (d) Quantification of cerebral infarct size at 24 hr after AAO. (e) Representative TTC-stained images of heart slices in AAO model. (f) Quantification of heart infarct size at 24 hr after AAO. Mean ± SD, *n* = 6.  ^*∗*^ ^*∗*^ ^*∗*^ ^*∗*^*p* < 0.0001. TTC, triphenyl tetrazolium chloride; MCAO, middle cerebral artery occlusion.

**Figure 3:**
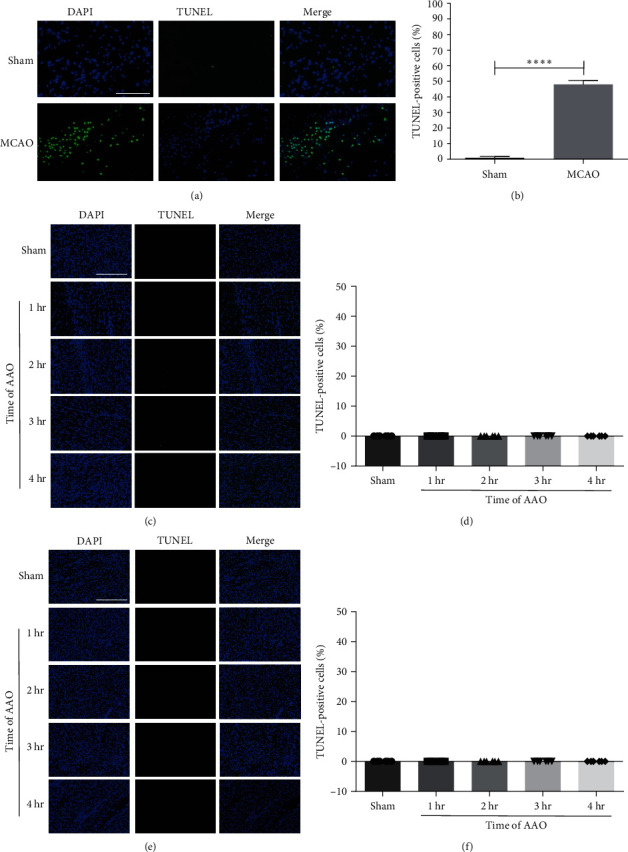
The effect of AAO on apoptosis of brain and heart in mice. The apoptotic cells of brain and heart slices were assessed by TUNEL staining. (a) Representative TUNEL-stained images of brain slices in MCAO model. (b) Quantification of TUNEL-positive cells at 24 hr after MCAO. (c) Representative TUNEL-stained images of hippocampus slices in AAO model. (d) Quantification of TUNEL-positive cells at 24 hr after AAO in brain. (e) Representative TUNEL-stained images of left ventricular slices in AAO model. (f) Quantification of TUNEL-positive cells at 24 hr after AAO in heart. Scale bar = 5 *μ*m. Mean ± SD, *n* = 6.  ^*∗*^ ^*∗*^ ^*∗*^ ^*∗*^*p* < 0.0001. TUNEL, terminal transferase-mediated dUTP nick-end labeling.

To investigate whether AAO-induced I/R indirectly results in ischemic injury to cardiac and neurologic tissue, we stained coronal sections of the heart and brain using TTC at 24 hr after reperfusion. TTC stain causes normal tissue to appear red, while necrotic, ischemic tissue appears pale (Figure 2(a)). Using the TTC stain, both cardiac and neurologic tissue stained red, indicating no ischemic injury in either area (Figure 2). To further investigate whether AAO-induced I/R causes cell apoptosis within the cardiac and neurologic tissues, TUNEL analysis was used to stain coronal sections of the heart and brain at 24 hr after reperfusion. The ischemic brain tissue directly supplied by the MCAO showed a positive TUNEL signal (Figures 3(a) and 3(b)). However, no TUNEL-positive cells were observed in neurologic or cardiac tissues (Figure 3(c)–3(f)).

### 3.2. The Effect of AAO on ROS Production in Brain and Heart

**Figure 4:**
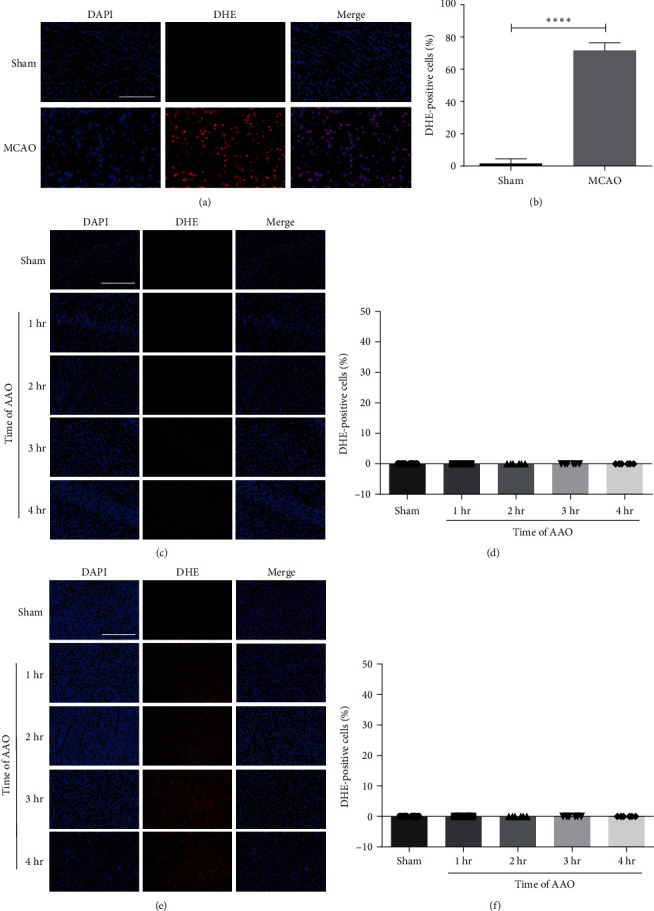
The effect of AAO on ROS production in brain and heart in mice. The ROS-damage cells in brain and heart slices were assessed by DHE staining. (a) Representative DHE-stained images of brain slices in MCAO model. (b) Quantification of DHE-positive cells at 24 hr after MCAO. (c) Representative DHE-stained images of hippocampus slices in AAO model. (d) Quantification of DHE-positive cells at 24 hr after AAO in brain. (e) Representative DHE-stained images of left ventricular slices in AAO model. (f) Quantification of DHE-positive cells at 24 hr after AAO in heart. Scale bar = 5 *μ*m. Mean ± SD, *n* = 6.  ^*∗*^ ^*∗*^ ^*∗*^ ^*∗*^*p* < 0.0001. ROS, reactive oxygen species; DHE, dihydroethidium.

Free radical production is a critical player in tissue destruction caused by reperfusion injury [8]. To determine whether AAO can cause oxidative stress induced by I/R, ROS production was assessed. DHE staining showed that I/R did not significantly increase ROS production compared to the sham group at 24 hr after reperfusion (Figure 4(c)–4(f)). However, MCAO increased ROS production in the brain (Figures 4(a) and 4(b)).

### 3.3. The Effect of AAO on Oxidant-Mediated Injury

**Figure 5:**
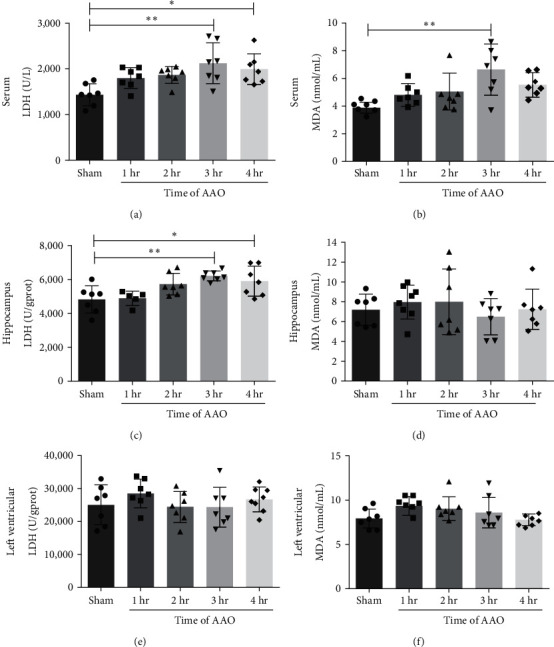
The expression level of LDH and MDA in serum, hippocampus, and left ventricular tissues of mice. The effect of AAO on LDH activity was evaluated using the Nanjing Jiancheng LDH kit. The effect of AAO on MDA activity was evaluated using the lipid peroxidation MDA assay kit. (a, b) Quantification of the LDH and MDA activity in serum samples. (c, d) Quantification of the LDH and MDA activity in hippocampus tissues. (e, f) Quantification of the LDH and MDA activity in left ventricular tissues. Mean ± SD, *n* = 7.  ^*∗*^*p* < 0.05,  ^*∗*^ ^*∗*^*p* < 0.01. LDH, lactate dehydrogenase; MDA, malondialdehyde.

To further analyze the effect of AAO on oxidant-mediated injury, levels of LDH and MDA were measured in the serum, hippocampus, and left ventricule at 24 hr after reperfusion. In the serum, LDH was significantly increased in the 3 and 4 hr groups compared to the sham group (*p* < 0.001 and *p* < 0.05, respectively, Figure 5(a)). MDA levels were significantly increased in the 3 hr group (*p* < 0.001, Figure 5(b)). In the hippocampus, LDH levels were increased in the 3 and 4 hr groups compared to the sham group (*p* < 0.001 and *p* < 0.05, respectively, Figure 5(c)). There were no significant differences in MDA levels among the groups (Figure 5(d)). In the left ventricle, there were no significant differences in LDH and MDA levels among the groups (Figures 5(e) and 5(f)).

### 3.4. The Effect of AAO on Antioxidant Capacity

**Figure 6:**
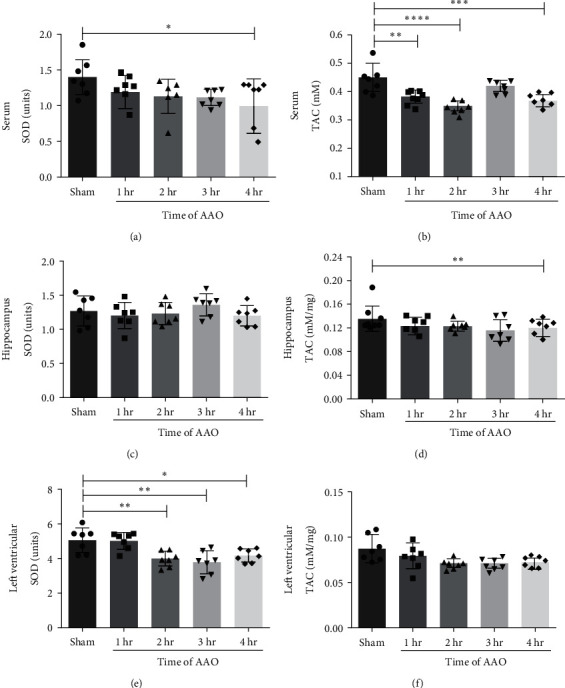
The expression levels of SOD and TAC in serum, hippocampus, and left ventricular tissues of mice. The effect of AAO on SOD activity was determined by SOD assay kit with WST-8. The effect of AAO on TAC activity was determined using T-AOC assay kit. (a, b) Quantification of the SOD and TAC activity in serum samples. (c, d) Quantification of the SOD and TAC activity in hippocampus tissues. (e, f) Quantification of the SOD and TAC activity in left ventricular tissues. Mean ± SD, *n* = 7.  ^*∗*^*p* < 0.05,  ^*∗*^ ^*∗*^*p* < 0.01,  ^*∗*^ ^*∗*^ ^*∗*^*p* < 0.001, and  ^*∗*^ ^*∗*^ ^*∗*^ ^*∗*^*p* < 0.0001. SOD, superoxide dismutase; TAC, total antioxidant capacity.

To determine the effect of AAO on antioxidant capacity, the levels of SOD and TAC were monitored in the serum, hippocampus, and left ventricle at 24 hr after reperfusion. AAO significantly decreased SOD and TAC levels of serum compared to the sham group (Figures 6(a) and 6(b)). However, changes in SOD and TAC levels were not statistically significant when comparing the AAO groups to each other. In the hippocampus, decreased TAC levels were observed in the 4 hr group compared to the sham group (*p* < 0.001, Figure 6(d)). In left ventricle, SOD levels were significantly decreased in 2–4 hr groups (*p* < 0.001, *p* < 0.001, and *p* < 0.05, respectively, Figure 6(e)).

### 3.5. The Effect of AAO on Proinflammatory and Anti-Inflammatory Factors

**Figure 7:**
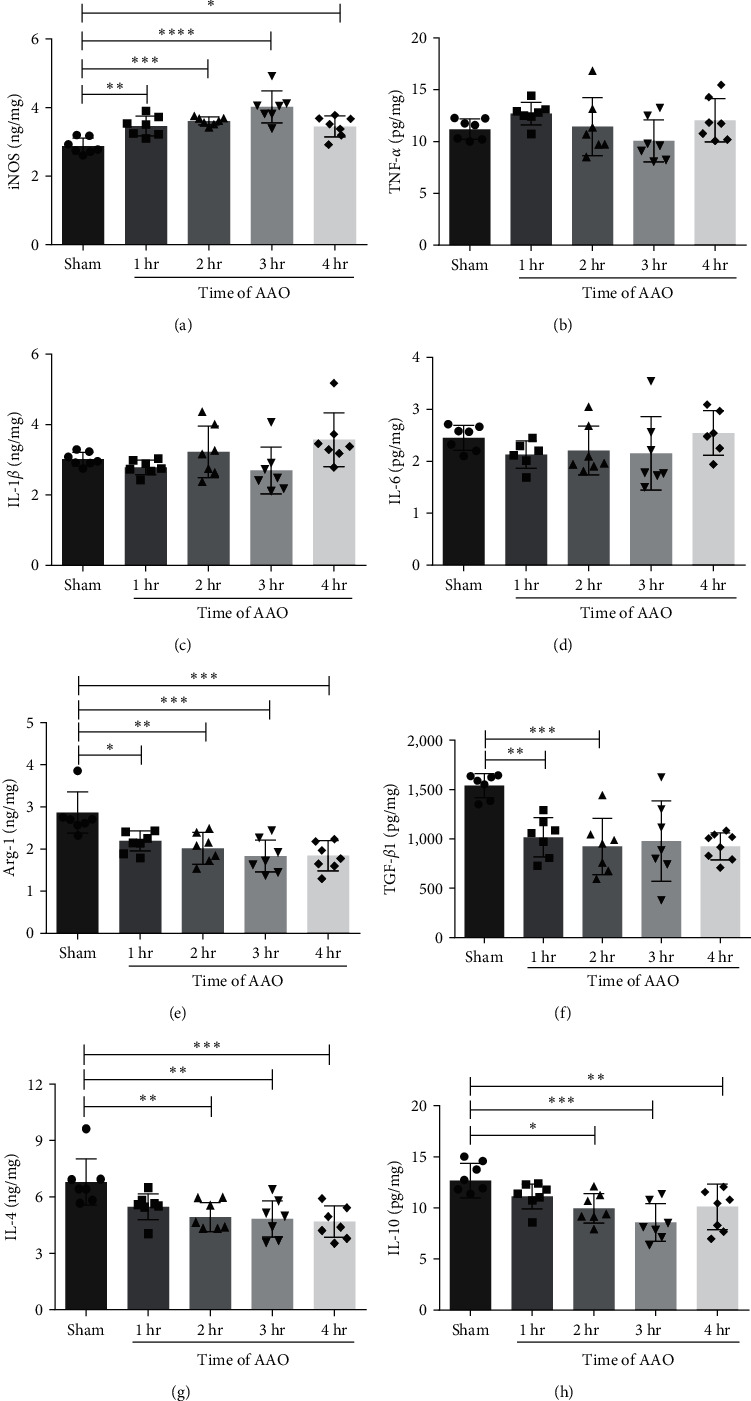
The concentration of iNOS, TNF-*α*, IL-1*β*, IL-6, Arg-1, TGF-*β*1, IL-4, and IL-10 in serum of mice. All mice of each group were sacrificed at 24 hr after AAO. The serum was collected and detected the concentration of proinflammatory and anti-inflammatory factors by ELISA assay. (a–h) Quantification of iNOS, TNF-*α*, IL-1*β*, IL-6, Arg-1, TGF-*β*1, IL-4, and IL-10 levels in serum samples. Mean ± SD, *n* = 7.  ^*∗*^*p* < 0.05,  ^*∗*^ ^*∗*^*p* < 0.01,  ^*∗*^ ^*∗*^ ^*∗*^*p* < 0.001, and  ^*∗*^ ^*∗*^ ^*∗*^ ^*∗*^*p* < 0.0001. ELISA, enzyme-linked immuno sorbent assay; iNOS, inducible nitric oxide synthase; TNF-*α*, tumor necrosis factor-*α*; IL-1*β*, interleukin-1*β*; IL-6, interleukin-6; Arg-1, Arginase-1; TGF-*β*1, transforming growth factor-beta 1; IL-4, interleukin-4; and IL-10, interleukin-10.

**Figure 8:**
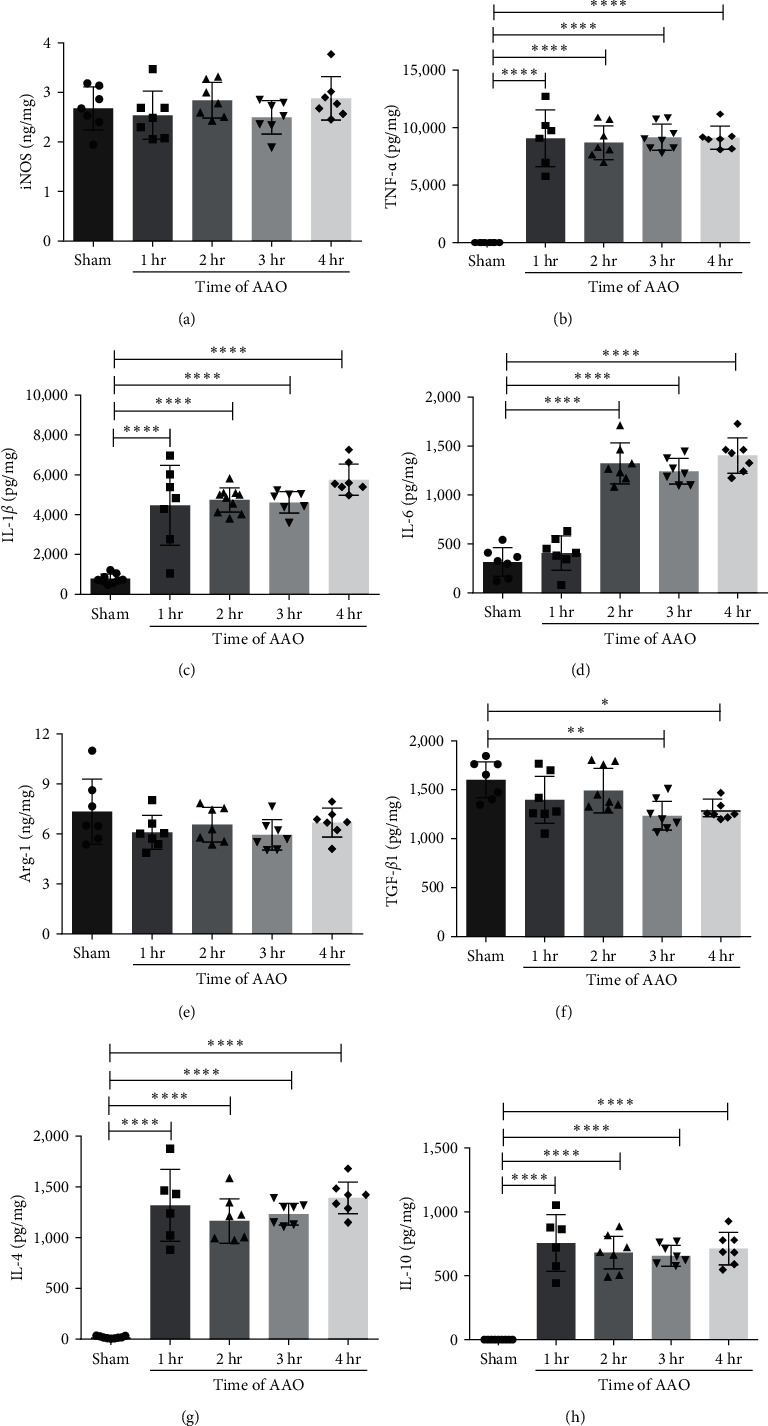
The concentration of iNOS, TNF-*α*, IL-1*β*, IL-6, Arg-1, TGF-*β*1, IL-4, and IL-10 in hippocampus tissue of mice. All mice of each group were sacrificed at 24 hr after AAO. The hippocampus tissue was isolated and detected the concentration of proinflammatory and anti-inflammatory factors by ELISA assay. (a–h) Quantification of iNOS, TNF-*α*, IL-1*β*, IL-6, Arg-1, TGF-*β*1, IL-4, and IL-10 levels in hippocampus tissues. Mean ± SD, *n* = 7.  ^*∗*^*p* < 0.05,  ^*∗*^ ^*∗*^*p* < 0.01, and  ^*∗*^ ^*∗*^ ^*∗*^ ^*∗*^*p* < 0.0001.

**Figure 9:**
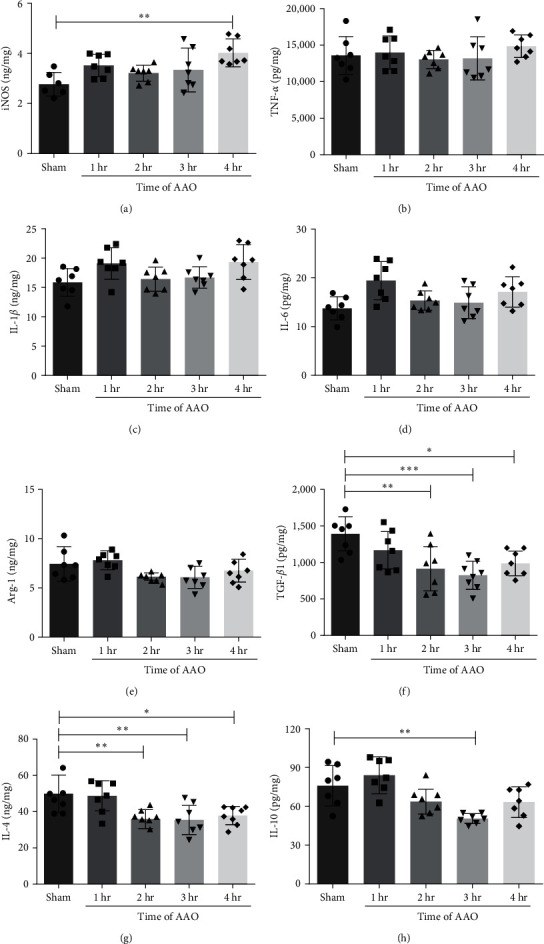
The concentration of iNOS, TNF-*α*, IL-1*β*, IL-6, Arg-1, TGF-*β*1, IL-4, and IL-10 in left ventricular tissue of mice. All mice were sacrificed at 24 hr after AAO. The left ventricular tissue was isolated and detected the concentration of proinflammatory and anti-inflammatory factors by ELISA assay. (a–h) Quantification of iNOS, TNF-*α*, IL-1*β*, IL-6, Arg-1, TGF-*β*1, IL-4, and IL-10 levels in left ventricular tissues. Mean ± SD, *n* = 7.  ^*∗*^*p* < 0.05,  ^*∗*^ ^*∗*^*p* < 0.01, and  ^*∗*^ ^*∗*^ ^*∗*^*p* < 0.001.

Studies have found that inflammatory cytokines directly released from site of injury can induce injury in distant organs [8]. In this study, we analyzed proinflammatory factors iNOS, TNF-*α*, IL-1*β*, and IL-6, and anti-inflammatory factors Arg-1, TGF-*β*1, IL-4, and IL-10 levels in the serum, hippocampus, and left ventricle at 24 hr after reperfusion. In the serum, iNOS significantly increased in all AAO groups when compared to the sham group (Figure 7(a)). There was no significant difference in proinflammatory factors TNF-*α*, IL-1*β*, and IL-6 levels among the groups (Figure 7(b)–7(d)). Anti-inflammatory factors Arg-1, TGF-*β*1, IL-4, and IL-10 were significantly decreased in all AAO groups compared to the sham group (Figure 7(e)–7(h)). In the hippocampus, levels of TNF-*α*, IL-1*β*, and IL-6 were significantly increased in the AAO groups compared to the sham group (*p* < 0.001, Figure 8(a)–8(d)). The level of TGF-*β*1 significantly decreased in the 3 and 4 hr groups. IL-4 and IL-10 levels significantly increased in all AAO groups compared to the sham group (*p* < 0.001, Figures 8(g) and 8(h)). In the left ventricle, iNOS significantly increased in the 4-hr group compared to the sham group (Figure 9(a)), and there were no significant differences in TNF-*α*, IL-1*β*, or IL-6 levels among the groups (*p* < 0.01, Figure 9(b)–9(d)). Anti-inflammatory factors TGF-*β*1, IL-4, and IL-10 were significantly decreased in all AAO groups when compared to the sham group (Figure 9(f)–9(h)).

## 4. Discussion

It has been reported that AAO-induced I/R can cause distal organ damage [7]. However, this is the first reported study to evaluate the effects of infrarenal AAO-induced I/R on neurologic and cardiac tissue. In this study, we found that varying lengths of infrarenal AAO (1–4 hr) followed by 24 hr of reperfusion had no effect on cell necrosis, cell apoptosis, or ROS production in neurologic and cardiac tissue. However, markers of tissue damage, such as LDH and MDA, and markers of antioxidant stress, such as SOD and TAC, had obvious changes in serum, hippocampal, and left ventricular samples. In addition to the oxidative stress-related biomarkers in all samples, some proinflammatory cytokines, namely iNOS TNF-*α*, IL-1*β*, and IL-6, were increased in the all three types of tissue. Some anti-inflammatory cytokines, particularly Arg-1, TGF-*β*1, IL-4, and IL-10, were decreased in serum and left ventricular tissues. Levels of IL-4 and IL-10 were significantly increased in the hippocampus.

While largely thought of in a pathological context, AAO is used as a clinical technique in a variety of surgical settings, such as massive abdominal bleeding hemostasis [24, 25]. However, ischemic injury to local and distal organs, as well as I/R injury after AAO should not be underestimated. Although longer blocking times better the hemostatic effect of AAO, the degree of tissue I/R injury may increase as the duration of ischemia is prolonged [26]. Therefore, it is imperative to identify a safe time window of ischemia in these settings. In this study, we investigated distant organ injury, particularly in the heart and brain, under varying lengths of AAO. We also assessed indicators of cell necrosis and apoptosis in the serum, finding no evidence of necrosis or apoptosis in either cardiac or hippocampal tissue in any AAO group. Findik et al. [17] reported that AAO-induced I/R (ischemia for 2 hr/reperfusion for 4 hr) significantly increased cell apoptosis in rat cardiac tissue. A recent report reinforced these findings, demonstrating that AAO-induced I/R (ischemia for 45 min/reperfusion for 7 days) also increased the cell apoptosis in rat cardiac tissue. The findings of both of these studies are inconsistent with our results [27]. This could possibly be attributed to differences in animal species. Different species may have different sensitivity to hypoxia/ischemia, and further research must be conducted to investigate these differences. Another study found that renal artery occlusion for 45 min/reperfusion for 24 hr did not result in cell apoptosis in rat neurologic tissue [10], which is supported by our findings. Although we did not find necrosis and apoptosis in neurologic and cardiac tissue, we did find significantly increased markers of tissue damage, specifically LDH, in hippocampal tissue in the 3 and 4 hr AAO groups. Despite lacking evidence of cell necrosis and apoptosis, increased levels of markers of cell damage, such as LDH [28] indicates that long-term ischemia has deleterious effects on hippocampal tissue. We also found increased levels of LDH the 3 and 4 hr AAO groups, supporting findings from previous studies [17, 28]. These results suggest that serum LDH can be used as a proxy to assess systemic damage caused by AAO.

Mounting evidences have shown that oxidative stress and inflammation play an critical role in systemic complications induced by AAO [28, 29]. Since the brain has the highest oxygen demand among any organ, it is extremely vulnerable to oxidative stress [30]. Both ischemia and reperfusion ramp up ROS production, a major contributor to I/R injury [31]. Levels of MDA are an indirect indicator free radical formation within the body [32]. Our study did not find increased ROS production in neurologic or cardiac tissue when probing with DHE, and increased MDA was only detected in serum. However, SOD and TAC levels, markers of antioxidant index capacity, were significantly decreased in the serum, hippocampus, and left ventricle. Future studies need to further investigate damage induced by oxidative stress using more sensitive and specific indicators.

In both clinical and animal models of I/R, various proinflammatory cytokines like iNOS, TNF-*α*, IL-1*β*, and IL-6 have been associated with tissue injury [33, 34]. Our study found increased serum and left ventricular iNOS. Chronic heart failure is associated with increased cytokine levels, which may trigger iNOS expression and the subsequent overproduction of cytotoxic NO [35]. AAO significantly increased levels TNF-*α*, IL-1*β*, and IL-6 in hippocampal tissue compared to the sham group (*p* < 0.0001). Anti-inflammatory factors IL-4 and IL-10 also significantly increased in the hippocampus in AAO groups compared to the sham group (*p* < 0.0001). IL-4 is an anti-inflammatory cytokine produced by a variety of immune cells [36]. IL-4 deficiency can acutely aggravate brain injury and neurological deficits after transient MCAO [37]. Multiple studies have shown that IL-10 acts as a neuroprotectant [38], as IL-10 deficient mice exhibit significantly increased infarction sizes [39]. In one experiment of ischemic stroke, IL-10 was present in both hemispheres of the brain [40]. In our study, AAO did not cause severe ischemic injury within the hippocampal region of the brain. We hypothesized that the body might activate such high expression of anti-inflammatory factors in an effort to protect neurologic tissue from inflammatory factors. Our results also showed that serum levels of anti-inflammation factors TGF-*β*, IL-4, and IL-10 were significantly decreased in AAO groups. While some studies have reported that in patients with abdominal aortic ligation after abdominal aortic aneurysm surgery, serum levels of these factors show a dynamic trend, increasing and decreasing postoperation, our results were not consistent with those findings. This could be because our model had only one reperfusion time point and did not trend these levels [41]. Also, the time of initiation and duration of reperfusion affect changes in cytokine levels [42]. Thus, future studies should further evaluate the dynamic nature of cytokine levels by trending these levels, rather than only measuring at one time point.

This study reinforced previous findings that AAO induces an inflammatory response that has deleterious effects on distant organs, in this case, the heart and brain. Although AAO did not induce cell necrosis and apoptosis in cardiac and neurologic tissue, inflammatory responses were well observed in serum, brain, and heart.

## Data Availability

The data used to support the findings of the current study are available from the corresponding author upon reasonable request.
